# Supplementary material for: Young mothers’ attitudes towards domestic violence and their maternal healthcare services utilization in Bangladesh: A multilevel cluster analysis
Source: PLoS One. 2022 Aug 1;17(8):e0268062. doi: 10.1371/journal.pone.0268062 (PMC9342752; doi:10.1371/journal.pone.0268062)
Supplement: Attachment — Submitted filename: PONE-D-21-14055 review ksa.pdf [file pone.0268062.s001.pdf]

PLOS ONE

Young mothers' attitudes towards domestic violence and their maternal healthcare services utilization in Bangladesh: A multilevel cluster analysis

--Manuscript Draft--

**Manuscript Number:**	PONE-D-21-14055
**Article Type:**	Research Article
**Full Title:**	Young mothers' attitudes towards domestic violence and their maternal healthcare services utilization in Bangladesh: A multilevel cluster analysis
**Short Title:**	Domestic violence and maternal healthcare services utilization in Bangladesh
**Corresponding Author:**	Md Rabiul Haque, PhD<br>Dhaka University<br>Dhaka, BANGLADESH
**Keywords:**	Antenatal care; Delivery care; domestic violence; Young mothers; Cluster analysis; Bangladesh
**Abstract:**	This paper examined the association between young mother's attitudes towards domestic violence and four or more antenatal care (ANC) and health-center-based delivery service utilization along with cluster variation using two waves of Bangladesh Demographic and Health Survey (2014 and 2018) data. Multilevel logistic regression results show that a strong cluster variation exists in four or more ANC and health-center-based delivery service utilization. Although the utilization of four or more ANC and health-center-based delivery services has increased over the years, it is far behind the targets of SDGs, particularly for young mothers with justified attitudes towards domestic violence. Extension of maternity allowance coverage and motivational programs are important policy recommendations.
**Order of Authors:**	Sawkia Afroz
	Tasmiah Sad Sutopa
	Md Rabiul Haque, PhD
**Additional Information:**	
**Question**	**Response**
**Financial Disclosure**<br><br>Enter a financial disclosure statement that describes the sources of funding for the work included in this submission. Review the submission guidelines for detailed requirements. View published research articles from *PLOS ONE* for specific examples.<br><br>This statement is required for submission and **will appear in the published article** if the submission is accepted. Please make sure it is accurate.	No - The funders had no role in study design, data collection and analysis, decision to publish, or preparation of the manuscript.

**Unfunded studies**<br>Enter: *The author(s) received no specific funding for this work.*<br><br>**Funded studies**<br>Enter a statement with the following details:<br>• Initials of the authors who received each award<br>• Grant numbers awarded to each author<br>• The full name of each funder<br>• URL of each funder website<br>• Did the sponsors or funders play any role in the study design, data collection and analysis, decision to publish, or preparation of the manuscript?<br>• **NO** - Include this sentence at the end of your statement: *The funders had no role in study design, data collection and analysis, decision to publish, or preparation of the manuscript.*<br>• **YES** - Specify the role(s) played.<br><br>* typeset	
**Competing Interests**<br><br>Use the instructions below to enter a competing interest statement for this submission. On behalf of all authors, disclose any competing interests that could be perceived to bias this work—acknowledging all financial support and any other relevant financial or non-financial competing interests.<br><br>This statement is required for submission and **will appear in the published article** if the submission is accepted. Please make sure it is accurate and that any funding sources listed in your Funding Information later in the submission form are also declared in your Financial Disclosure statement.<br><br>View published research articles from *PLOS ONE* for specific examples.	The authors have declared that no competing interests exist.

Powered by Editorial Manager® and ProduXion Manager® from Aries Systems Corporation

**NO authors have competing interests**<br><br>Enter: *The authors have declared that no competing interests exist.*<br><br>**Authors with competing interests**<br><br>Enter competing interest details beginning with this statement:<br><br>*I have read the journal's policy and the authors of this manuscript have the following competing interests: [insert competing interests here]*<br><br>* typeset	
**Ethics Statement**<br><br>Enter an ethics statement for this submission. This statement is required if the study involved:<br><br>• Human participants<br>• Human specimens or tissue<br>• Vertebrate animals or cephalopods<br>• Vertebrate embryos or tissues<br>• Field research<br><br>Write "N/A" if the submission does not require an ethics statement.<br><br>General guidance is provided below. Consult the submission guidelines for detailed instructions. **Make sure that all information entered here is included in the Methods section of the manuscript.**	We used secondary data which is available for public use. Thus ethical clearance is not required for this study.

**Format for specific study types**<br><br>**Human Subject Research (involving human participants and/or tissue)**<br>• Give the name of the institutional review board or ethics committee that approved the study<br>• Include the approval number and/or a statement indicating approval of this research<br>• Indicate the form of consent obtained (written/oral) or the reason that consent was not obtained (e.g. the data were analyzed anonymously)<br><br>**Animal Research (involving vertebrate animals, embryos or tissues)**<br>• Provide the name of the Institutional Animal Care and Use Committee (IACUC) or other relevant ethics board that reviewed the study protocol, and indicate whether they approved this research or granted a formal waiver of ethical approval<br>• Include an approval number if one was obtained<br>• If the study involved *non-human primates*, add *additional details* about animal welfare and steps taken to ameliorate suffering<br>• If anesthesia, euthanasia, or any kind of animal sacrifice is part of the study, include briefly which substances and/or methods were applied<br><br>**Field Research**<br><br>Include the following details if this study involves the collection of plant, animal, or other materials from a natural setting:<br>• Field permit number<br>• Name of the institution or relevant body that granted permission	
**Data Availability**<br><br>Authors are required to make all data underlying the findings described fully available, without restriction, and from the time of publication. PLOS allows rare exceptions to address legal and ethical concerns. See the PLOS Data Policy and FAQ for detailed information.	Yes - all data are fully available without restriction

A Data Availability Statement describing where the data can be found is required at submission. Your answers to this question constitute the Data Availability Statement and **will be published in the article**, if accepted.<br><br>**Important:** Stating 'data available on request from the author' is not sufficient. If your data are only available upon request, select 'No' for the first question and explain your exceptional situation in the text box.<br><br>Do the authors confirm that all data underlying the findings described in their manuscript are fully available without restriction?	
**Describe where the data may be found in full sentences. If you are copying our sample text, replace any instances of XXX with the appropriate details.**<br><br>• If the data are **held or will be held in a public repository**, include URLs, accession numbers or DOIs. If this information will only be available after acceptance, indicate this by ticking the box below. For example: *All XXX files are available from the XXX database (accession number(s) XXX, XXX.).*<br>• If the data are all contained **within the manuscript and/or Supporting Information files**, enter the following: *All relevant data are within the manuscript and its Supporting Information files.*<br>• If neither of these applies but you are able to provide **details of access elsewhere**, with or without limitations, please do so. For example:<br><br>*Data cannot be shared publicly because of [XXX]. Data are available from the XXX Institutional Data Access / Ethics Committee (contact via XXX) for researchers who meet the criteria for access to confidential data.*<br><br>*The data underlying the results presented in the study are available from (include the name of the third party*	Data files for this survey are open to access and use through the DHS program website. USAID. The Demographic and Health Survey ICF, Rockville,Washington, D.C., USA: United States Agency for International Development; 2020 [cited 2020 8 august]. Available from: https://dhsprogram.com/data/available-datasets.cfm?fbclid=IwAR1H7zsRnId8cmR2kwd8fD-p2XDP7gQrXAd-oOOzKQdHiq7VpLUMDPLxM2c.

Powered by Editorial Manager® and ProduXion Manager® from Aries Systems Corporation

and contact information or URL).<br>• This text is appropriate if the data are owned by a third party and authors do not have permission to share the data.<br><br>* typeset	
Additional data availability information:	

Powered by Editorial Manager® and ProduXion Manager® from Aries Systems Corporation

Manuscript

Click here to access/download;Manuscript;Manuscript_Domestic violence and MCH service in Bangladesh.docx

Young mothers' attitudes towards domestic violence and their maternal healthcare services utilization in Bangladesh: A multilevel cluster analysis

Sawkia Afroz[1], Tasmiah Sad Sutopa[2], and Md Rabiul Haque[3]

[1] Lecturer, Department of Population Sciences, University of Dhaka, Bangladesh

[2] Lecturer, Department of Statistics, University of Dhaka, Bangladesh

[3] Professor, Department of Population Sciences, University of Dhaka, Bangladesh

Email Addresses:
Sawkia.afroz@du.ac.bd
Ts_sutopa@du.ac.bd
Rabiuldps@du.ac.bd

Corresponding Author:
Md Rabiul Haque, PhD
Professor, Department of Population Sciences,
Faculty of Social Sciences,
University of Dhaka,
Dhaka 1000
Bangladesh
Email: Rabiuldps@du.ac.bd
Phone: +88 01705531207

Declaration of interest: None.
Acknowledgment of Funding: There was no financial support for this study.

Young mothers' attitudes towards domestic violence and their maternal healthcare services utilization in Bangladesh: A multilevel cluster analysis

Abstract

This paper examined the association between young mother's attitudes towards domestic violence and four or more antenatal care (ANC) and health-center-based delivery service utilization along with cluster variation using two waves of Bangladesh Demographic and Health Survey (2014 and 2018) data. Multilevel logistic regression results show that a strong cluster variation exists in four or more ANC and health-center-based delivery service utilization. Although the utilization of four or more ANC and health-center-based delivery services has increased over the years, it is far behind the targets of SDGs, particularly for young mothers with justified attitudes towards domestic violence. Extension of maternity allowance coverage and motivational programs are important policy recommendations.

Keywords: Antenatal care, Delivery care, Domestic violence, Young mothers, Cluster analysis, Bangladesh

Introduction

Assurance of healthy lives for all is one of the commitments of the Sustainable Development Goals (SDGs), the blueprint for building a better world for the next generation. In achieving equity in the health systems, a reduction in maternal mortality is greatly desirable which can be ensured through access to and use of quality antenatal care (ANC) and health-center-based delivery care. Utilization of four or more ANC and health-center-based delivery care is an important WHO-recommended strategies to reduce preventable maternal mortalities and their morbidities (1-3). Moreover, young mothers have a greater risk to experience maternal mortality due to their limited access to four or more ANC and health-center-based delivery care compared to adult mothers (4-7). Previous studies have identified several barriers of four or more ANC and health-center-based delivery service utilization related to demographic and socio-economic aspects of the population. Apart from these, the association of behavioral factors, especially the attitudes of young mothers towards domestic violence and access to four or more ANC and health-center-based delivery care need to be explored as it has become a burning issue over the world now-a-days. The international community has also been committed to ending all forms of violence against all women and girls by 2030 under SDG-5 (Target 5.2) (8).

In 2019, countries from all around the world participating in the International Conference on Population and Development (ICPD+25) initiated an integrated approach to attain triple zero in terms of unmet need for contraception, preventable maternal deaths, and gender-based violence and harmful practices (9). Globally, 35.0% of women have endured sexual or physical violence by their intimate and or non-intimate partner (10). Around 23.2% of women from wealthy countries and 37.7% of women from South East Asian countries including Bangladesh have experienced various forms of violence at different stages of their life-cycle (11). Violence against women adversely affects their physical and mental health because most abused women are less capable to care for themselves and their offspring (12, 13). Evidence also suggested that in developing countries like Bangladesh domestics violence against women during pregnancy decreased their consumption of nutritious foods which in the long run affect their pregnancy outcomes by increasing the likelihood of miscarriage, preterm birth, stillbirth, underweight at birth, and deaths followed by maternal mortality and morbidity (4, 14-23). Domestic violence against women causes

disempowerment, particularly for young women (24, 25) which ultimately limits their access to four or more ANC and health-center-based delivery care.

Bangladesh government has been implementing several initiatives such as a community-based skilled birth attendant program, maternity allowance (MA) program, basic and comprehensive emergency obstetric care, active management of pregnancy complications, establish women's friendly hospital, etc. to improve maternal healthcare services utilization, targeting to reach 50% four or more ANC and 65% health-center-based delivery by 2022 (26) and four or more ANC and health-center-based delivery 98% by 2030 (27). Under the MA program, each selected poor woman received an amount of TK 800.0 equivalent to 9.52 US$ per month for three years since gestation to purchase health care and nutrient foods for themselves and also for their children (28). Although the utilization rate has increased in 2017-18 (referred to 2018 onward) compared to 2014, yet only 47% of mothers in Bangladesh received four or more ANC and 49% of them delivered their last child's birth at a health-center in 2018 (29). Such a pattern of using four or more ANC and health-center-based delivery care in Bangladesh is largely associated with women's lower social position, lower level of education and financial condition, and inadequate sense about their rights (30-33). While the unavailability, inaccessibility, and unaffordability are strongly correlated with lower utilization of four or more ANC and health-center-based delivery services among Bangladeshi women, the association of domestic violence with antenatal and delivery care services utilization is less studied in the context of Bangladesh (31, 34-36).

Socio-cultural norms and gender roles typically shape Bangladeshi women's domestic violence accepting attitudes (36, 37). In Bangladesh, at least three in every five women experienced physical or sexual violence and one in five women justified wife-beating by their husbands (32, 38-40). Therefore, understanding the effects of domestic violence on young mothers' four or more ANC and health-center-based delivery service utilization is important for developing effective maternal healthcare policies and interventions.

The association between domestic violence and the lower utilization of four or more ANC and health-center-based delivery care receives less attention previously. Earlier studies focused on either utilization of any ANC or violence caused by an intimate partner (36). Moreover, the

situation of ANC and health-center-based delivery care for young mothers, particularly those aged 15-24 years, remains unexplored as previous studies mostly considered women of all age groups (25, 36-38). Also, the variations in the use of four or more ANC and health-center-based delivery by clusters are not considered in the past literature. In the context of Bangladesh, neither has existing studies considered domestic violence towards young mothers and its implication on using four or more ANC and health-center-based delivery care nor appropriate statistical models have been used to reveal the cluster variations (41). Moreover, to our knowledge, no such studies yet used the recent two waves of BDHS data (2018 and 2014) for comparing the utilization of four or more ANC and health-center-based delivery services by women's attitudes towards domestic violence in Bangladesh. The findings of this study may contribute developing countries like Bangladesh, which contains a large portion (28%) of ever-married young women (29). More specifically, a better understanding of the effect of several covariates along with cluster variation which is examined in this study may assist the policymakers to execute the intervention programs targeting to increase the utilization of WHO-recommended four or more ANC and health-center-based delivery services for young mothers in a planned way.

Materials and Methods

Data Source

The Demographic Health Survey (DHS) was performed in 85 developing nations including Bangladesh measuring the progress of population health and nutritional status. Since 1993, this survey has been conducted in Bangladesh in three-year intervals by the National Institute of Population Research and Training (NIPORT) of the Ministry of Health and Family Welfare (39). Data files for this survey are open to access and use through the DHS program website (42). This study used data from the recent two waves of Bangladesh Demographic and Health Surveys (BDHS), 2014 and 2018 (29, 39).

Sampling design

The BDHS was guided by a two-stage stratified cluster sampling for the household survey (29, 39). The sampling framework of this survey was developed using the total list of enumeration areas (EAs) covering the entire country of the most recent census developed by the Bangladesh Bureau of Statistics (BBS). EAs are defined as geographic areas having an average of 120

households in each (43). In the first stage, EAs were selected with probability proportional to the size of EA. In the second stage, using a systemic sampling procedure a certain number of households on average were selected from each EA with an equal probability to provide statistically valid estimates of health-related national-level indicators within the subgroups of divisions and place of residence. Ever-married women aged 15-49 years were interviewed for this survey to evaluate the utilization pattern of maternal healthcare services. The details of the sampling design of BDHS 2014 and BDHS 2018 are described elsewhere (29, 39).

Study Population

This study considered only currently married young mothers, aged 15-24 years who had a recent live birth in three years preceding the survey (44). This allowed 2431 observations from BDHS 2014 and 2599 observations from BDHS 2018, after extracting and cleaning data available for the study. Among the studied young mothers, 32.4% of them in BDHS 2014 whereas 48.3% among them in BDHS 2018 took four or more ANC visits. Moreover, 40.1% of young mothers delivered their recent birth at a health-center in BDHS 2014 and 51.7% of them received health-center-based delivery care in BDHS 2018.

Variables' Characteristics

Outcome variables

The study considered two binary outcome variables as indicators of maternal healthcare services utilization. The first outcome variable, utilization of four or more ANC visits categorized into two: 'yes' refers to those who had four or more ANC visits, and 'no' refers to those who took zero to three ANC visits. The second outcome variable is the place of delivery (PoD) which indicates whether the births were delivered at home or under a health center. If the birth took place at any health center, it is recorded as 'health center-based delivery' and if the birth was delivered at home then it is recorded as 'home-based delivery'.

Independent variables

The prime focus of this study is on the independent variable 'young mothers' attitude toward domestic violence'. A composite variable was measured through young mothers' attitudes towards justification of wife-beating or hitting by their husbands, which was grounded by their responses

to five questions: 1) if she burns the food, 2) if she argues with husband, 3) if she goes out without telling husband, 4) if she neglects the children, and 5) if she refuses to have sexual intercourse with husband. However, this variable for analysis purposes was coded into two categories: (1) ‘favorable’ refers to those who justified domestic violence with at least one of the above reasons, and (2) ‘opposed’ refers to otherwise.

A range of socio-economic, and demographic variables was also included in the multivariate analysis. The variable region was coded into seven divisions for BDHS 2014: (1) Barisal, (2) Chittagong, (3) Dhaka, (4) Khulna, (5) Rajshahi, (6) Rangpur, and (7) Sylhet (39). Mymensing is added as another administrative area along with these divisions in BDHS 2018(29). Place of residence was coded into two: (1) rural, and (2) urban. Households’ wealth index was coded into three: (1) poor, (2) middle, and (3) rich. Educational status of young mothers and their husbands was coded into three categories: (1) no education, (2) primary who completed grade five, and (3) secondary who completed grade 10, and (4) higher who attained above the secondary level education. The respondent is described to have access to the media if she read a newspaper or magazine/watched television/listened to the radio. Media exposure was classified into two: (1) ‘yes’ and (2) ‘no’. Young mothers’ decision-making capacity was coded into two: (1) ‘yes’, and (2) ‘no’. If any respondent took part in making decisions solely or partially at least one of the issues related to own healthcare, key household purchases, and visit her family members or relatives are categorized as “yes” in young mothers’ autonomy variable and vice versa. The working status of mothers was categorized as (1) employed, and (2) unemployed. Birth order was classified into two: (1) first birth, and (2) others.

Statistical Analyses

As the BDHS survey follows a two-stage stratified clustering sampling procedure, the two-level multilevel logistic model uses to examine the cluster effect among young mothers, and the random effect component is introduced for each cluster as the young mothers are nested into the clusters (45, 46). The measures of association between two outcome variables and covariates are assessed through chi-square tests. The independent variables that were significantly associated with the outcome variables are included in the regression model. For each of the two outcome variables,

two models are considered- a null model without any explanatory variables and a complete model with all covariates.

Suppose Y_{ij} be the binary outcome variable for j^{th}individual in i^{th} cluster where $j = 1, 2, \dots, n_i$ and $i = 1, 2, \dots, k$. Let $x_{ij} = (x_{ij1}, x_{ij2}, \dots, x_{ijp})'$ be the $p \times 1$ vector of independent variables for j^{th}individual in i^{th} cluster and $\beta = \left(\beta_1, \beta_2, \dots, \beta_p\right)'$ be the $p \times 1$ vector of regression parameters. Then multilevel logistic regression can be written as

$$\ln \frac{\pi_{ij}}{1 - \pi_{ij}} = x'_{ij}\beta + u_i$$

Where $\pi_{ij} = E\left(Y_{ij} \middle| u_i\right)$ and u_i is random intercept where$u_i \sim N\ (0, {\sigma_u}^2)$. In this setup, the fixed effect of covariates can be expressed through adjusted odds ratio, and random effect can be revealed through intra-cluster correlation (ICC) which means correlation among individuals within the same cluster. The denoted ρ can be measured by the variance component of random intercept that is $\rho = \frac{{\sigma_u}^2}{{\sigma_u}^2 + \frac{\pi^2}{3}}$(47).

Results

The distribution of young mothers aged 15-24 years by background characteristics presented in Table 1 shows that the percentage of young mothers with four or more ANC visits was increased by about 16% (32.4% in 2014 and 48.3% in 2018) and health-center-based delivery was increased by about 12% (40.1% in 2014 and 51.7% in 2018) over the years. Young mothers' opposing attitudes towards domestic violence was also increased by 9.4%, from 72.7% in 2014 to 82.1% in 2018. Whilst, the proportion of poor young mothers was increased over the years, the proportion of rich young mothers was reduced. The percentage of young mothers and their husbands had no education was decreased and a higher level of education for both of them was increased between 2014 and 2018. The percentage of young mothers with access to media was also increased over the years. Moreover, young mothers' decision-making capacity was increased by 11.5% and the proportion of employed young mothers was increased by about 15% between 2014 and 2018. The proportion of first birth order was higher for young mothers in both years.

Table 1: Distribution of several characteristics of young mothers aged 15-24 years in Bangladesh, BDHS 2014 and 2018

**Characteristics**	**BDHS 2014 (n=2431)**	**BDHS 2018 (n=2599)**
At least 4 ANC		
Yes	32.4	48.3
No	67.6	51.7
Place of Delivery (PoD)		
Health-center-based	40.1	51.7
Home-based	59.9	48.3
Attitude towards domestic violence		
Favorable	27.3	17.9
Opposed	72.7	82.1
Division		
Barisal	11.8	11.1
Chattogram	19.5	16.7
Dhaka	16.6	14.4
Khulna	12.4	10.7
Rajshahi	12.9	10.9
Rangpur	13.0	11.0
Sylhet	13.9	13.1
Mymensingh[*]	-	12.2
Place of residence		
Rural	68.3	67.7
Urban	31.7	32.3
Wealth index		
Poor	40.4	43.1
Middle	19.7	19.0
Rich	39.9	37.9
Young mothers' educational level		
No Education	7.2	3.6
Primary	27.9	25.4
Secondary	54.5	52.1
Higher	10.3	18.9
Husband's educational level		
No Education	35.2	30.3
Primary	45.0	46.1
Secondary	7.2	6.0
Higher	12.6	17.6
Access to media		
No	36.3	35.8
Yes	63.7	64.2
Decision-making capacity		
No	31.8	20.3
Yes	68.2	79.7
Working status		
No	83.2	68.5
Yes	16.8	31.5
Birth order		
Otherwise	35.2	37.6
First birth	64.8	62.4

[*] Mymensingh division has been included in BDHS 2018 wave as a separate division.

The overall utilization rates of four or more ANC visits and health-center-based delivery care among young mothers was increased between 2014 and 2018. However, the utilization rate was strikingly low for young mothers who had justified attitudes towards the reasons for domestic violence than those who had opposite attitudes, which indicate the negative effects of domestic violence on young mothers' utilization of four or more ANC and health-center-based delivery care (Table 2). It also shows that four or more ANC (29.5% in 2014 and 41.8% in 2018) and health-center-based delivery (30.4% in 2014 and 38.2 % in 2018) service utilization rates among young mothers who had justified attitudes towards domestic violence if a woman goes out without telling her husband were far behind the targets of Bangladesh's 4th Health, Population and Nutrition Sector Program (HPNSP) and Sustainable Development Goals (SDGs). Though the utilization rates of four or more ANC and health-center-based delivery care for young mothers who had justified attitudes towards the other reasons of domestic violence such as: if she neglects the children, argues with her husband, refuses to have sex with her husband, and burnt food were increased in 2018 than that were in 2014 but it tended to lag behind the aforementioned targets.

Table 2: Percentage distribution of four or more ANC and health-center based delivery utilization among young mothers who have recent birth by reasons for domestic violence according to BDHS 2014 and 2018

**Domestic violence by husband is justified if women**	**% received at least 4 ANC**		**% delivered birth at a health-center**	
	**BDHS 2014**	**BDHS 2018**	**BDHS 2014**	**BDHS 2018**
Goes out without telling husband	29.5	41.8	30.4	38.2
Neglects the children	29.1	41.0	30.7	42.2
Argues with husband	30.1	37.2	28.4	41.9
Refuses to have sex with husband	24.5	26.3	22.0	36.8
Burnt food	21.6	30.4	19.3	39.1

The percentage distribution of young mothers' who received four or more ANC and health-center-based delivery care by demographic and socioeconomic characteristics of the respondents is presented in Table 3. The table shows that young mothers' utilization of four or more ANC and health-center-based delivery care were varied substantially by their attitudes towards domestic violence in all categories, except for ANC use in 2014. Young mothers who had favorable attitudes towards domestic violence were significantly less likely to utilize four or more ANC compared to their counterparts who had opposed attitudes (p-value<0.001), particularly in 2018. Between 2014 and 2018, the utilization rate of four or more ANC was increased 10.5% among young mothers who had a favorable attitude towards domestic violence and 16.8% who had an opposite attitude towards such violence. However, over the same period, the rate of health-center-based delivery

care was increased more among young mothers who had favorable attitudes towards domestic violence (13.4%) than those who had opposite attitudes (9.8%).

Table 3: Young mothers aged 15-24 years who received four or more ANC and health-center-based delivery by demographic and socioeconomic attributes, BDHS 2014 and 2018

**Variables**	**Received four or more ANC**				**Delivered birth at a health center**			
	**BDHS 2014**		**BDHS 2018**		**BDHS 2014**		**BDHS 2018**	
	**%**	**p-value**	**%**	**p-value**	**%**	**p-value**	**%**	**p-value**
Attitude toward domestic violence								
Favorable	30.1	0.146	40.6	<0.001	30.6	<0.001	44.0	<0.001
Opposed	33.2		50.0		43.6		53.4	
Division								
Barisal	26.6	<0.001	37.8	<0.001	27.6	<0.001	44.8	<0.001
Chattogram	28.1		42.3		37.8		49.7	
Dhaka	37.2		50.7		47.6		58.1	
Khulna	39.1		62.1		57.0		64.3	
Rajshahi	30.7		48.1		46.6		57.2	
Rangpur	43.0		52.3		39.2		54.2	
Sylhet	23.1		60.8		24.3		42.8	
Mymensingh	-		37.5		-		44.3	
Place of residence								
Rural	26.6	<0.001	43.2	<0.001	33.3	<0.001	47.7	<0.001
Urban	44.9		59.0		54.7		60.0	
Wealth index								
Poor	20.1	<0.001	37.1	<0.001	22.8	<0.001	36.4	<0.001
Middle	27.7		46.7		38.8		50.9	
Rich	47.2		61.9		58.2		69.4	
Young mothers' educational level								
No Education	11.9	<0.001	20.2	<0.001	21.0	<0.001	25.5	<0.001
Primary	22.7		34.3		28.0		33.7	
Secondary	35.7		51.3		43.5		53.7	
Higher	55.4		64.4		67.7		75.4	
Husband's educational level								
No Education	21.8	<0.001	35.5	<0.001	25.8	<0.001	36.3	<0.001
Primary	33.8		47.5		41.2		50.7	
Secondary	41.7		55.4		54.3		63.7	
Higher	51.6		70.2		67.6		76.6	
Access to media								
No	20.2	<0.001	34.3	<0.001	23.4	<0.001	36.1	<0.001
Yes	39.3		56.2		49.6		60.4	
Decision-making capacity								
No	30.3	0.130	46.0	0.242	39.5	0.689	48.7	0.130
Yes	33.4		48.9		40.3		52.4	
Working status								
No	33.4	0.014	48.3	1.000	42.1	<0.001	55.3	<0.001
Yes	27.2		48.3		29.9		43.8	
Birth order								
Otherwise	26.9	<0.001	41.6	<0.001	28.6	<0.001	39.8	<0.001
First birth	35.4		52.4		46.3		58.8	

There was a significant geographical variation in using four or more ANC and health-center-based delivery services (Table 3). In terms of using health-center-based delivery Sylhet division had been identified as a low-performing area of all divisions for both of the study points (24.3% and 42.8% respectively). Though Sylhet was a low-performing area considering the percentage of four or more ANC utilization in 2014 (23.1%), the situation became worsen for Mymensing in 2018 among all divisions (37.5%). Between 2014 and 2018, rural young women experienced a greater increase in using four or more ANC (16.6%) and health-facility-based delivery care (14.4%) than their counterparts' urban young women, 14.1% and 5.3% respectively. A subclass analysis by wealth status suggests that poor young women were less likely to use four or more ANC and health-facility-based delivery care than rich young women. The highest growth in receiving four or more ANC was noticed among young mothers from the middle wealth quintile by 19.0% (27.7% to 46.7%) followed by poor 17.0% (20.1% to 37.1%) and rich 14.7% (47.2% to 61.9%) between 2014 and 2018. However, the increase in the proportion of health-center-based delivery care was highest among poor young mothers followed by middle and rich young mothers.

Moreover, young mothers and their husbands with higher educational status were significantly more likely to use four or more ANC and health-center-based delivery care than those who had no education (Table 3). Whilst young mothers from all subclass of education were experienced greater use of four or more ANC and health-center-based delivery care between 2014 and 2018, the lowest increase occurred among young mothers with no education, 8.3%, and 4.5% respectively. Also, the utilization of four or more ANC and health-center-based delivery care were found substantially higher for young mothers who had access to media than those who had no such access, in both years. Between 2014 and 2018, four or more ANC and health-center-based delivery care utilization was increased by 14.1% and 12.7% respectively for young mothers with access to media. Surprisingly, employed young mothers compared to unemployed were significantly less prevalent in using health-center-based delivery care in both years. Young mothers were used four or more ANC and health-center-based delivery care more for their first birth compared to any other births. However, there was a 14.7% increase of four or more ANC (26.9% to 41.6%) and an 11.2% increase in health-facility-based delivery care (28.6% to 39.8%) in terms of the births other than the first birth between the two survey waves. However, women's decision-making capacity was not found significant in any of the years for both of the outcomes.

Table 4: Fixed effect of several factors and random effects obtained from multilevel logistic regression model on at least 4 ANC and health-center-based delivery utilization

**Variables**	**At least 4 ANC**				**Delivered birth at a health center**			
	**BDHS 2014**		**BDHS 2018**		**BDHS 2014**		**BDHS 2018**	
	**AOR**	**p-value**	**AOR**	**p-value**	**AOR**	**p-value**	**AOR**	**p-value**
Attitude toward domestic violence								
Opposed	1	-	1	-	1	-	1	-
Favorable	1.11	0.380	0.795	0.065	0.668	0.001	0.803	0.064
Division								
Barisal	1	-	1	-	1	-	1	-
Chittagong	0.743	0.214	0.978	0.919	1.326	0.207	0.861	0.444
Dhaka	1.159	0.536	1.094	0.697	1.915	0.004	1.030	0.886
Khulna	1.415	0.162	2.310	<0.001	3.706	<0.001	1.753	0.008
Rajshahi	0.947	0.828	1.737	0.021	2.509	<0.001	1.453	0.077
Rangpur	2.371	<0.001	3.114	<0.001	1.948	0.005	1.687	0.013
Sylhet	0.876	0.611	1.044	0.857	0.980	0.936	0.924	0.702
Mymensingh	-	-	1.695	0.024	-	-	1.033	0.876
Place of residence								
Rural	1	-	1	-	1	-	1	-
Urban	1.741	<0.001	1.674	<0.001	1.728	<0.001	1.106	0.382
Wealth index								
Poor	1	-	1	-	1	-	1	-
Middle	1.215	0.217	1.128	0.375	1.430	0.015	1.212	0.136
Rich	2.240	<0.001	1.621	0.001	2.140	<0.001	2.198	<0.001
Young mothers' educational level								
No Education	1	-	1	-	1	-	1	-
Primary	1.917	0.019	2.079	0.017	1.048	0.842	1.247	0.421
Secondary	2.852	<0.001	3.158	<0.001	1.283	0.287	1.951	0.014
Higher	4.920	<0.001	3.241	<0.001	1.861	0.035	2.873	<0.001
Husband's educational level								
No Education	1	-	1	-	1	-	1	-
Primary	1.183	0.195	1.363	0.008	1.360	0.013	1.199	0.099
Secondary	1.194	0.416	1.496	0.062	1.601	0.027	1.460	0.072
Higher	1.398	0.094	2.702	<0.001	2.419	<0.001	2.117	<0.001
Access to media								
No	1	-	1	-	1	-	1	-
Yes	1.261	0.089	1.638	<0.001	1.409	0.008	1.566	<0.001
Working status								
No	1	-	-	-	1	-	1	-
Yes	0.846	0.316	-	-	0.631	<0.001	0.731	0.003
Birth order								
Otherwise	1	-	1	-	1	-	1	-
First birth	1.120	0.252	1.181	0.093	1.727	0.001	1.667	<0.001
**Intra-cluster Correlation (ICC)**								
Null model	0.25		0.24		0.16		0.20	
Full model	0.16		0.15		0.27		0.07	

From the adjusted covariates (Table 4), the lower utilization of healthcare facilities for antenatal and delivery care was found strongly correlated with young mothers' positive attitudes towards domestic violence in both of the survey years. More specifically, young mothers who had a favorable attitude towards domestic violence had 33.2% lower odds of getting health-center-based

delivery care (OR=0.668) in 2014 whereas the odds was lowered by 19.7% in 2018 (OR= 0.803). The utilization of four or more ANC and health-center-based delivery services significantly varied by division. Compared to other divisions, Rangpur experienced greater progress in the utilization of four or more ANC between 2014 (OR 2.371) and 2018 (OR 3.114). However, Khulna division was experienced the highest odds of receiving health-center-based delivery both in 2014 (OR 3.706) and 2018 (OR 1.753). In contrast, the lowest odds (≤1) was found for Chittagong among all divisions in both periods in terms of using four or ANC and health-center-based delivery care. Moreover, young mothers residing in urban areas were more likely to utilize four or more ANC in both periods than their counterparts in rural young women. However, the urban-rural difference was significantly reduced in using four or more ANC and health-center-based delivery care over the years.

The odds ratio of four or more ANC and health-center-based delivery care was increased with improved wealth status (Table 4). The same pattern in odds ratio was found by young mothers' and their husbands' educational attainment. These made the fact vivid that household wealth status and higher educational attainment of mothers and their husbands increased their utilization of four or more ANC and health-center-based delivery care. Moreover, access to media had a positive effect in using four or more ANC and health-center-based delivery services. Between 2014 and 2018, the likelihood of receiving four or more ANC and delivering birth at a health-center was increased among young mothers with access to media, from (ORs) 1.261 to 1.638 and 1.409 to 1.566 respectively. Surprisingly, working young mothers were less likely to use health-center-based delivery care (OR= 0.631 and 0.731) in both years than their counterparts non-working young mothers. Moreover, there were 72.7% and 66.7% higher odds of having a health-center-based delivery care in 2014 and 2018 respectively for the first birth of young mothers than the other births.

Table 4 also reported the cluster effect on four or more ANC and place of delivery service utilization based on the random effect for both of the survey years. Firstly, a separate null model was applied for each of the outcome variables to check whether the application of the multilevel logistic model in this study is justified. The result shows cluster variances for both outcome variables. The ICC obtained from the null model in 2018 data for four or more ANC was 0.24

which indicates that 24.0% of the total variation in receiving four or more ANC related to variation between clusters which was slightly low from 2014 BDHS (ICC=0.25). On a contrary, the ICC estimated for the place of delivery from the null model was 0.20 which anticipated 20.0% of the total variation in place of delivery was attributable to the clusters where mothers were residing which was higher than that of 2014 (ICC= 0.16). There was a radical decrease in ICCs obtained separately for both dependent variables from the final model. The cluster variation observed in the null model can mostly be explained by consideration of the effect of independent variables on four more ANC and health-center-based delivery services.

Discussion

This paper uniquely identifies the strong association between domestic violence and the utilization of four or more ANC and health-center-based delivery care along with the presence of cluster variation in Bangladesh. This pattern should be of major concern to achieve universal maternal healthcare coverage, as inequity and domestic violence towards women are important barriers indeed in the use of four or more ANC and health-center-based delivery care. For the first time for Bangladesh to our knowledge, this study uses the most recent BDHS 2018 data along with the data of BDHS 2014 in analyzing the effects of domestic violence on young mothers' four or more ANC and health-center-based delivery care. The overall utilization of four or more ANC and health-center-based delivery care among young mothers has increased by 10.5% and 13.4% respectively in 2018 than that was in 2014. However, the healthcare service utilization for four or more ANC and health-center-based delivery care is found substantially low for young mothers who had justified attitudes towards several reasons of domestic violence. Moreover, the utilization rate of four or more ANC and health-center-based delivery care among young mothers is far behind the targets of 4th HPNSP and SDGs.

The study demonstrates that the utilization of four or more ANC and health-center-based delivery services were increased less over the years among young mothers who had favorable attitudes towards domestic violence than those who had the opposite attitudes. The limited use of four or more ANC in 2014 and health-center-based delivery care in 2014 and 2018 was strongly associated with young mothers' positive attitude towards domestic violence. These findings are broadly found consistent with studies conducted in Asian and African country contexts (21, 22, 48-50). Positive attitudes towards domestic violence of some women are likely to be associated with their inferior

position, negligence and devaluation, submissive nature towards husbands, poor educational status, lack of self-esteem, lower sense of entitlement, lack of awareness about rights, traditional gender norms and practices in Bangladesh (24, 35, 38, 51-53). Therefore, domestic violence is an important social as well as public health concern in improving healthcare service utilization for quality ANC and health-center-based delivery care in Bangladesh and some other countries like India, Tanzania, Ethiopia, etc.

Moreover, this study found the presence of cluster variation in utilization of four or more ANC and health-center-based delivery care for both of the survey years which indicates that young mothers of underprivileged communities have inadequate utilization of four or more ANC and health-center-based delivery care. This finding is largely found consistent with a previous study conducted in Ethiopia which reported that mothers who are members of communities with high amenities have greater access to and utilization of healthcare services for WHO-recommended number of ANC visits and hospital-based delivery care than those who live in communities with low amenities. The variations in utilization of four or more ANC and health-center-based delivery services among clusters due to geographic, cultural, and socio-economic differences is another public health concern in Bangladesh that needs to be addressed to make healthcare services more accessible to all women.

The present study suggests that along with domestic violence, multiple socio-economic and demographic covariates are significantly associated with the lower utilization of four or more ANC and health-center-based delivery care. Geographical variation is also found significant for reducing the use of four or more ANC and health-center-based delivery care. Young mothers of rural areas are less likely to utilize four or more ANC and health-center-based delivery care compared to those who are urban residents which is consistent with earlier studies (31, 33, 35, 54). The utilization of four or more ANC and health-center-based delivery services significantly varies with divisions. Further, the utilization rate of four or more ANC among young mothers of Sylhet division has increased remarkably in 2018 which was previously identified as low performing division in terms of using ANC visits (29, 39). However, among the divisions, young mothers of Sylhet division are less likely to utilize health-center-based delivery care which is found consistent with previous studies (55, 56). Possible causes are poor transportation, geographical remoteness, and inadequate

counseling about the importance of health-center-based delivery care (Kamal et al., 2013; Shabnam, 2011).

Our study in line with other study identified that poor young mothers are found less likely to utilize four or more ANC and health-center-based delivery care compared to rich young mothers in both survey periods (35, 40, 57). This finding highlights the fact that despite the socio-economic progress, still Bangladesh is experiencing inequities in the utilization of healthcare services (35). Unlike other studies, we also found that the utilization of four or more ANC and health-center-based delivery care is found associated with young mothers' education and their husband's education which is also identical with earlier studies conducted in Bangladesh (31, 35). Both young mothers and their husbands with a higher level of educational status appear with more awareness and knowledge regarding quality maternal healthcare which increase young mothers' likelihood of using that services for antenatal and delivery care (35, 48, 58, 59). Mass media exposure also contributes to increase young mothers' utilization of four or more ANC and health-center-based delivery care which is also reported in previous studies (31, 36, 58). Mass media is a great source of diffusing information that increases awareness of individuals through behavior change communication and develops a tendency among them to adopt a positive or new behavior.

Surprisingly, employed young mothers in our study are found less likely to utilize four or more ANC and health-center-based delivery in both of the survey years. Despite being employed a few Bangladeshi young women have the liberty to exercise their own decision and ability to spend money for their healthcare. Besides due to shyness, scarcity of female healthcare practitioners, and restriction imposed by husbands and family members many employed young mothers sometimes feel uncomfortable using the WHO-required number of ANC and hospital-based delivery care (16, 23, 33, 38, 51, 60). This study also reveals young mothers with first pregnancy are more likely to utilize four or more ANC and health-center-based delivery care compared to their other births. First pregnancy may be viewed with delicacy due to the new experience and excitement of the married couples which increases their utilization of ANC and health-center-based delivery care, especially for young mothers (33, 54, 61).

Conclusions

The findings of the study demonstrate that though the utilization of four or more ANC and health-center-based delivery care has increased over the years, young mothers with justified attitudes towards domestic violence are less likely to use of four or more ANC and health-center-based delivery care than mothers with opposite attitudes. A strong variation in the clusters, which is identified in our study, is also associated with lower utilization of health services for four or more ANC and health-center-based delivery care. In view of the lower utilization of health services for ANC and delivery care among mothers who experienced domestic violence, the health system of Bangladesh may initiate social campaigning and counseling intervention for young women, their husbands, and other family members highlighting the benefits of using the WHO-recommended four or more ANC and health-center-based delivery care as well as on the health consequences of domestic violence. In light of the variation among clusters, the coverage of the maternity allowance program should be extended, especially in low performing communities, for universal maternal healthcare coverage in Bangladesh (28).

The major strength of our study is the consideration of nationally representative recent two waves of BDHS data (2014 and 2018) to identify the significant relationship between attitudes towards domestic violence and utilization of four or more ANC and health-center-based delivery care among young Bangladeshi women. Moreover, the cluster variations in utilization of four or more ANC and health-center-based delivery care has been assessed rigorously in this study. Our findings suggest that there is a scope of further study to identify the cluster variations in terms of using four or more ANC and health-center-based delivery care considering the community-level features and extending the analysis to a three-level multilevel analysis. The study may have recall bias though the effect of recall bias is minimized by considering the young mothers only who had their last birth in the preceding three years of the surveys. This study using regression outputs identified the associations between the predictors and outcomes measures only, not the causal relationship.

Acknowledgments

We are indebted to ICF international, Rockville, Maryland, USA for giving us with access to the 2014 and 2017-2018 Bangladesh Demographic and Health Survey datasets. We would like to thank the editor and the anonymous reviewers for their constructive insights and guidelines.

Conflict of Interest

We declared no conflict of interest.

Author Contributions

Conceptualization: Sawkia Afroz, Tasmiah Sad Sutopa, Md. Rabiul Haque.

Data curation: Tasmiah Sad Sutopa

Formal analysis: Tasmiah Sad Sutopa, Sawkia Afroz.

Methodology: Tasmiah Sad Sutopa, Md. Rabiul Haque.

Writing the original draft: Sawkia Afroz, Tasmiah Sad Sutopa, Md. Rabiul Haque,

Writing, reviewing and editing: Md. Rabiul Haque, Sawkia Afroz, Tasmiah Sad Sutopa.

Supervision: Md. Rabiul Haque.

References:

1. Bhutta ZA, Darmstadt GL, Hasan BS, Haws RA. Community-based interventions for improving perinatal and neonatal health outcomes in developing countries: a review of the evidence. Pediatrics. 2005;115(Supplement 2):519-617.
2. WHO. Standards for improving quality of maternal and newborn care in health facilities. World Health Organization, 2016 9241511214.
3. Ruiz-Cantero MT, Guijarro-Garvi M, Bean DR, Martínez-Riera JR, Fernández-Sáez J. Governance commitment to reduce maternal mortality. A political determinant beyond the wealth of the countries. Health & place. 2019;57:313-20.
4. Reynolds HW, Wong EL, Tucker H. Adolescents' use of maternal and child health services in developing countries. International family planning perspectives. 2006:6-16.
5. Temin M, Levine R. Start with a girl: a new agenda for global health: Center for global development; 2009.
6. Kavitha N. Are young mothers in India deprived of maternal health care services? a comparative study of urban and rural areas. Journal of Health Management. 2015;17(2):204-20.
7. Dyalchand A, Pande RP, Kulkarni G, Khale M. Improving use of maternal care services among married adolescent girls: an intervention study in rural India. Journal of Biosocial Science. 2020:1-20.
8. UN. TRANSFORMING OUR WORLD: the 2030 agenda for sustainable development goals. Geneva: United Nations, 2015.
9. UNFPA. Nairobi Statement on ICPD25: Accelerating the Promise. New York, United States United Nations Population Fund, 2019.
10. WHO. Violence against women [Fact sheet]. Geneva:World Health Organizations, 2017.
11. WHO. Global and regional estimates of violence against women: prevalence and health effects of intimate partner violence and non-partner sexual violence: World Health Organization; 2013.

12. Ellsberg M, Jansen HA, Heise L, Watts CH, Garcia-Moreno C. Intimate partner violence and women's physical and mental health in the WHO multi-country study on women's health and domestic violence: an observational study. The lancet. 2008;371(9619):1165-72.
13. van Wormer K, Link RJ. Social work and social welfare: A human rights Foundation: Oxford University Press; 2018.
14. Curry M, Perrin N, Wall E. Effects of abuse on maternal complications and birth weight in adult and adolescent women. Obstetrics & gynecology. 1998;92(4):530-4.
15. Silverman JG, Gupta J, Decker MR, Kapur N, Raj A. Intimate partner violence and unwanted pregnancy, miscarriage, induced abortion, and stillbirth among a national sample of Bangladeshi women. BJOG: An International Journal of Obstetrics & Gynaecology. 2007;114(10):1246-52.
16. Shah PS, Shah J. Maternal exposure to domestic violence and pregnancy and birth outcomes: a systematic review and meta-analyses. Journal of women's health. 2010;19(11):2017-31.
17. Seng JS, Low LK, Sperlich M, Ronis DL, Liberzon I. Post- traumatic stress disorder, child abuse history, birthweight and gestational age: a prospective cohort study. BJOG: An International Journal of Obstetrics & Gynaecology. 2011;118(11):1329-39.
18. Campbell JC. Health consequences of intimate partner violence. The lancet. 2002;359(9314):1331-6.
19. Koski AD, Stephenson R, Koenig MR. Physical violence by partner during pregnancy and use of prenatal care in rural India. Journal of health, population, and nutrition. 2011;29(3):245.
20. Leserman J, Li Z, Drossman D, Hu YJ. Selected symptoms associated with sexual and physical abuse history among female patients with gastrointestinal disorders: the impact on subsequent health care visits. Psychological medicine. 1998;28(2):417-25.
21. Stephenson R, Koenig MA, Acharya R, Roy TK. Domestic violence, contraceptive use, and unwanted pregnancy in rural India. Studies in family planning. 2008;39(3):177-86.
22. Stephenson R, Koenig MA, Ahmed S. Domestic violence and symptoms of gynecologic morbidity among women in North India. International family planning perspectives. 2006:201-8.
23. Sarkar N. The impact of intimate partner violence on women's reproductive health and pregnancy outcome. Journal of Obstetrics and Gynaecology. 2008;28(3):266-71.
24. Akanda L, Shamim I. Women and violence: a comparative study of rural and urban violence against women in Bangladesh: Women for Women; 1985.
25. Ameen N. Wife abuse in Bangladesh: An unrecognised offence: The University Press Dhaka; 2005.
26. MOHFW. 4th Health, Population and Nutrition Sector Programme (4th HPNSP). Dhaka, Bangladesh: Ministry of Health and Family Welfare (MOHFW), 2017.
27. NIPORT. Bangladesh Demographic and Health Survey 2014 : Policy Brief Dhaka, Bangladesh and Rockville, Maryland, USA: National Institute of Population Research and Training (NIPORT) and ICF International, 2016.
28. MOWCA. Maternity Allowance and Working Lactating Mother Allowance. In: Cabinet Division and the General Economics Division (GED) PC, editor. Dhaka, Bangladesh: Ministry of Women and Children Affairs,Goverment of Peoples Republic of Bangladesh; 2018.
29. NIPORT, ACPR, ICF, Inc. I. Bangladesh Demographic and Health Survey 2017-18. National Institute of Population Research and Traning 2020.
30. Alam MS, Tareque MI, Peet ED, Rahman MM, Mahmud T. Female participation in household decision making and the justification of wife beating in Bangladesh. Journal of interpersonal violence. 2018:0886260518772111.
31. Haque SE, Rahman M, Mostofa MG, Zahan MS. Reproductive health care utilization among young mothers in Bangladesh: does autonomy matter? Women's Health Issues. 2012;22(2):e171-e80.
32. ICDDRB. From evidence to policy: addressing gender-based violence gainst women and girls in Bangladesh. The icddr, b, Dhaka, Bangladesh. 2013.
33. Haque MR, Parr N, Muhidin S. The effects of household's climate-related displacement on delivery and postnatal care service utilization in rural Bangladesh. Social Science & Medicine. 2020;247:112819.

34. Rahman MH, Mosley WH, Ahmed S, Akhter HH. Does service accessibility reduce socioeconomic differentials in maternity care seeking? Evidence from rural Bangladesh. Journal of biosocial science. 2008;40(1):19-33.
35. Amin R, Shah NM, Becker S. Socioeconomic factors differentiating maternal and child health-seeking behavior in rural Bangladesh: A cross-sectional analysis. International journal for equity in health. 2010;9(1):9.
36. Rahman M, Nakamura K, Seino K, Kizuki M. Intimate partner violence and use of reproductive health services among married women: evidence from a national Bangladeshi sample. BMC public health. 2012;12(1):913.
37. Khan MN, Islam MM. Women's attitude towards wife-beating and its relationship with reproductive healthcare seeking behavior: A countrywide population survey in Bangladesh. PLoS one. 2018;13(6):e0198833.
38. Tareque MI, Alam MS, Peet ED, Rahman MM, Rahman KM. Justification of wife beating and utilization of antenatal and delivery care in Bangladesh. Journal of interpersonal violence. 2020:0886260519898444.
39. NIPORT, ACPR, ICF, Inc. I. Bangladesh demographic and health survey 2014. National Institute of Population Research and Traning 2016.
40. Rahman KF. Focus on domestic violence in Bangladesh: a study from criminological perspectives. Journal of international women's studies. 2019;20(3):98-115.
41. Johnston HB, Naved RT. Spousal violence in Bangladesh: a call for a public-health response. Journal of health, population, and nutrition. 2008;26(3):366.
42. USAID. The Demographic and Health Survey ICF, Rockville,Washington, D.C., USA: United States Agency for International Development; 2020 [cited 2020 8 august]. Available from: https://dhsprogram.com/data/available-datasets.cfm?fbclid=IwAR1H7zsRnId8cmR2kwd8fD-p2XDP7gQrXAd-oOOzKQdHiq7VpLUMDPLxM2c.
43. BBS. Bangladesh population and housing census 2011. Bangladesh Bureau of Statistics Dhaka, Bangladesh; 2011.
44. WHO. Adolescent health in the South-East Asia Region. Geneva, World Health Organization: 2017.
45. Gilmour A, Anderson R, Rae A. The analysis of binomial data by a generalized linear mixed model. Biometrika. 1985;72(3):593-9.
46. Agresti A. Categorical data analysis: John Wiley & Sons; 2003.
47. Rodriguez G, Elo I. Intra-class correlation in random-effects models for binary data. The Stata Journal. 2003;3(1):32-46.
48. Tiruye TY, Harris ML, Chojenta C, Holliday E, Loxton D. Determinants of intimate partner violence against women in Ethiopia: A multi-level analysis. PLoS one. 2020;15(4):e0232217.
49. Kabir R, Majumder AA, Arafat SY, Chodwhury RK, Sultana S, Ahmed SM, et al. Impact of Intimate Partner violence on ever married women and utilization of antenatal care services in Tanzania. Journal of College of Medical Sciences-Nepal. 2018;14(1):7-13.
50. Musa A, Chojenta C, Geleto A, Loxton D. The associations between intimate partner violence and maternal health care service utilization: a systematic review and meta-analysis. BMC Women's Health. 2019;19(1):36.
51. Adegoke TG, Oladeji D. Community norms and cultural attitudes and beliefs factors influencing violence against women of reproductive age in Nigeria. Editorial Advisory Board e. 2005;20(2):265-73.
52. Puri M, Frost M, Tamang J, Lamichhane P, Shah I. The prevalence and determinants of sexual violence against young married women by husbands in rural Nepal. BMC research notes. 2012;5(1):291.
53. Koenig MA, Ahmed S, Hossain MB, Mozumder AKA. Women’s status and domestic violence in rural Bangladesh: individual-and community-level effects. Demography. 2003;40(2):269-88.
54. Haque MR, Parr N, Muhidin S. Climate-Related Displacement and Antenatal Care Service Utilization in Rural Bangladesh. International Perspectives on Sexual and Reproductive Health. 2020;46:175-85.

55. Pulok MH, Uddin J, Enemark U, Hossin MZ. Socioeconomic inequality in maternal healthcare: An analysis of regional variation in Bangladesh. Health & place. 2018;52:205-14.
56. Nonyane BA, Kazmi N, Koffi AK, Begum N, Ahmed S, Baqui AH, et al. Factors associated with delay in care–seeking for fatal neonatal illness in the Sylhet district of Bangladesh: results from a verbal and social autopsy study. Journal of global health. 2016;6(1).
57. Bloom SS, Wypij D, Gupta MD. Dimensions of women's autonomy and the influence on maternal health care utilization in a north Indian city. Demography. 2001;38(1):67-78.
58. Navaneetham K, Dharmalingam A. Utilization of maternal health care services in Southern India. Social science & medicine. 2002;55(10):1849-69.
59. Mai V, Phyu WE. Intimate partner violence and utilization of reproductive and maternal health services in Cambodia. Journal of Health Research. 2019.
60. Pennington A, Orton L, Nayak S, Ring A, Petticrew M, Sowden A, et al. The health impacts of women's low control in their living environment: A theory-based systematic review of observational studies in societies with profound gender discrimination. Health & place. 2018;51:1-10.
61. Haque MR, Parr N, Muhidin S. Parents' healthcare-seeking behavior for their children among the climate-related displaced population of rural Bangladesh. Social Science & Medicine. 2019;226:9-20.
