# Supplementary material for: Young mothers’ attitudes towards domestic violence and their maternal healthcare services utilization in Bangladesh: A multilevel cluster analysis
Source: PLoS One. 2022 Aug 1;17(8):e0268062. doi: 10.1371/journal.pone.0268062 (PMC9342752; doi:10.1371/journal.pone.0268062)
Supplement: Attachment — Submitted filename: Response to Reviewers_PONE-D-21-14055_R2.docx [file pone.0268062.s003.docx]

**Responses to Reviewers**

We wish to thank to the reviewers for their constructive feedback. We reproduce the reviews below point-by-point and summarize our response to each point immediately followed by the point.

1. If the authors have adequately addressed your comments raised in a previous round of review and you feel that this manuscript is now acceptable for publication, you may indicate that here to bypass the “Comments to the Author” section, enter your conflict of interest statement in the “Confidential to Editor” section, and submit your "Accept" recommendation.

**Comment from Reviewer #2:** All comments have been addressed

2. Is the manuscript technically sound, and do the data support the conclusions?

**Comment from Reviewer #2:** Yes

3. Has the statistical analysis been performed appropriately and rigorously?

**Comment from Reviewer #2:** Yes

4. Have the authors made all data underlying the findings in their manuscript fully available?

**Comment from Reviewer #2:** Yes

5. Is the manuscript presented in an intelligible fashion and written in Standard English?

**Comment from Reviewer #2**: No

**Response to the reviewer:** Language of the manuscript has been copyedited. Texts in some places have also been modified making the sentences clearer and avoiding duplication.

6. Review Comments to the Author. Please use the space provided to explain your answers to the questions above. You may also include additional comments for the author, including concerns about dual publication, research ethics, or publication ethics. (Please upload your review as an attachment if it exceeds 20,000 characters)

**Comment from Reviewer #2**: First, I want to thank the authors for the changes they made in the revised version. However, I still think the English of the manuscript is poor and needs revision. The authors are suggested to increase clarity of their writing by revising it with a native speaker or an expert user of English or a professional grammar editing software/service.

**Response to the reviewer:** Thanks for spotting the language errors. We have gone through the manuscript rigorously and copyedited the whole manuscript to increase the clarity of the writing.
